# Transthoracic placement of fiducials with ultrasound or electronic navigational bronchoscopy needle guidance by the interventional pulmonologist: A case series

**DOI:** 10.1002/rcr2.818

**Published:** 2021-07-29

**Authors:** Dany Gaspard, Ziad Boujaoude, Gregory Kubicek, Wissam Abouzgheib

**Affiliations:** ^1^ Division of Pulmonary and Critical Care Medicine Cooper University Hospital Camden New Jersey USA; ^2^ Division of Radiation Oncology Cooper University Hospital Camden New Jersey USA

**Keywords:** electronic navigational bronchoscopy, fiducial placement, stereotactic body radiotherapy, ultrasound

## Abstract

Stereotactic body radiotherapy (SBRT) has become one of the main options for treatment of thoracic malignancies, leading to the need for more fiducial marker placement. We report cases where these fiducials were placed transthoracically by interventional pulmonologists using ultrasound (US) and electronic navigational bronchoscopy (ENB) needle guidance. Six cases were identified in the Cooper University Hospital medical records where such procedures were performed, alone or in combination with other interventions. All six patients underwent successful placement of fiducials. Concomitant bronchoscopic procedures were performed in four cases. All patients proceeded to SBRT without the need for further interventions. The overall retention rate of fiducials was 80%. No complications were noted. Fiducials' placement by interventional pulmonologists using US or ENB needle guidance is safe and effective, and may be combined with other procedures in a single setting.

## Graphical abstract

In this study, we aim to evaluate the feasibility and safety of transthoracic ultrasound or electronic navigational bronchoscopy (ENB) needle‐guided fiducial placement in the chest performed by interventional pulmonologists in the bronchoscopy suite. We describe six such cases performed at Cooper University Hospital in Camden, New Jersey, USA.

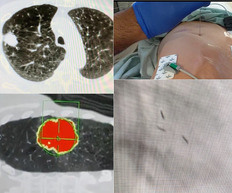

## INTRODUCTION

Thoracic malignancies have increased in prevalence over the past few years, leading to a myriad of new treatment modalities. Among those, stereotactic radiation surgery (also referenced as stereotactic body radiotherapy [SBRT]) has become one of the main therapeutic options.^1^

This has led to an increased need for placement of fiducial markers. Fiducial markers are small gold or carbon markers placed bronchoscopically or transthoracically in close proximity to the tumour in order to track its movement with respiration (lung masses can move as much as 2 cm with breathing movements) and deliver radiation beams directly to it while minimizing the exposure of surrounding healthy tissues.

Fiducials are most commonly placed bronchoscopically or transthoracically using computed tomography (CT) guidance. Placement using ultrasound (US) guidance has also been described but very few reports are published on the subject and none address thoracic malignancies in particular.

In the past few years, multiple reports of fiducial placement using electronic navigational bronchoscopy (ENB) were published.^2^, ^3^, ^4^ ENB are systems that match a CT scan image of the patient's chest with the patient's anatomy and guide the bronchoscopist through the complex bronchial system and into the mass being sampled. Some ENB systems also offer the option of ENB‐guided needle transthoracic guidance for sampling of lung masses with excellent yield.^5^ However, use of this technique for fiducial placement has not been described.

In this study, we aim to evaluate the feasibility and safety of transthoracic US or ENB needle‐guided fiducial placement in the chest performed by interventional pulmonologists in the bronchoscopy suite. We describe six such cases performed at Cooper University Hospital in Camden, New Jersey, USA.

## CASE SERIES

### Methods

Records of the Rowan University/Cooper University Hospital were reviewed between June 2015 and June 2017 looking for placement of fiducial markers transthoracically using either US or ENB needle.

Patients were adults >18 years of age. Each case was discussed individually by a multidisciplinary team of oncologists, pulmonologists, radiation oncologists, radiologists and pathologists, and a decision was made to proceed with biopsy followed by radiation therapy. Surgical resection was considered for oligometastatic cases but the operative risk was considered prohibitively high because of mediastinal location/proximity to the heart and great vessels in one patient and poor functional status in two other patients. For cases with multiple metastatic lesions, targeting of a specific metastasis was done because of increased standardized uptake value (SUV) uptake on Positron Emission Tomography (PET) scan for one single metastasis (and significant decrease in SUV uptake for the other lesions) in one patient, and increase in size of a metastatic lesion with regression of the other lesions on follow‐up imaging for another patient after chemotherapy.

In these specific cases, a gating technique for SBRT was planned, and fiducial placement was recommended by the radiation oncologist. A decision was made to perform the biopsies and the fiducial placement during the same procedure. Other interventions (such as bronchoscopy, endobronchial US, etc.) were also allowed during the same intervention.

For each patient, age, sex, smoking status and underlying malignancy were noted. In addition, we noted the location of the mass, distance from pleura, details of the procedure, number of fiducials placed, number of fiducials retained and distance from tumour during radiation planning.

#### Description of the procedure

Using either direct US visualization or ENB needle guidance, an 18‐ or 20‐gauge core needle was introduced into the mass. Core needle biopsies were obtained. Then, one or multiple Gold Fiducial markers were introduced into the core needle and pushed into the tumour using the core needle's trochar. US was used to visualize the fiducial inside the tumour when possible. Fluoroscopy was also used to assess for retention or migration of the placed fiducials.

**FIGURE 1:**
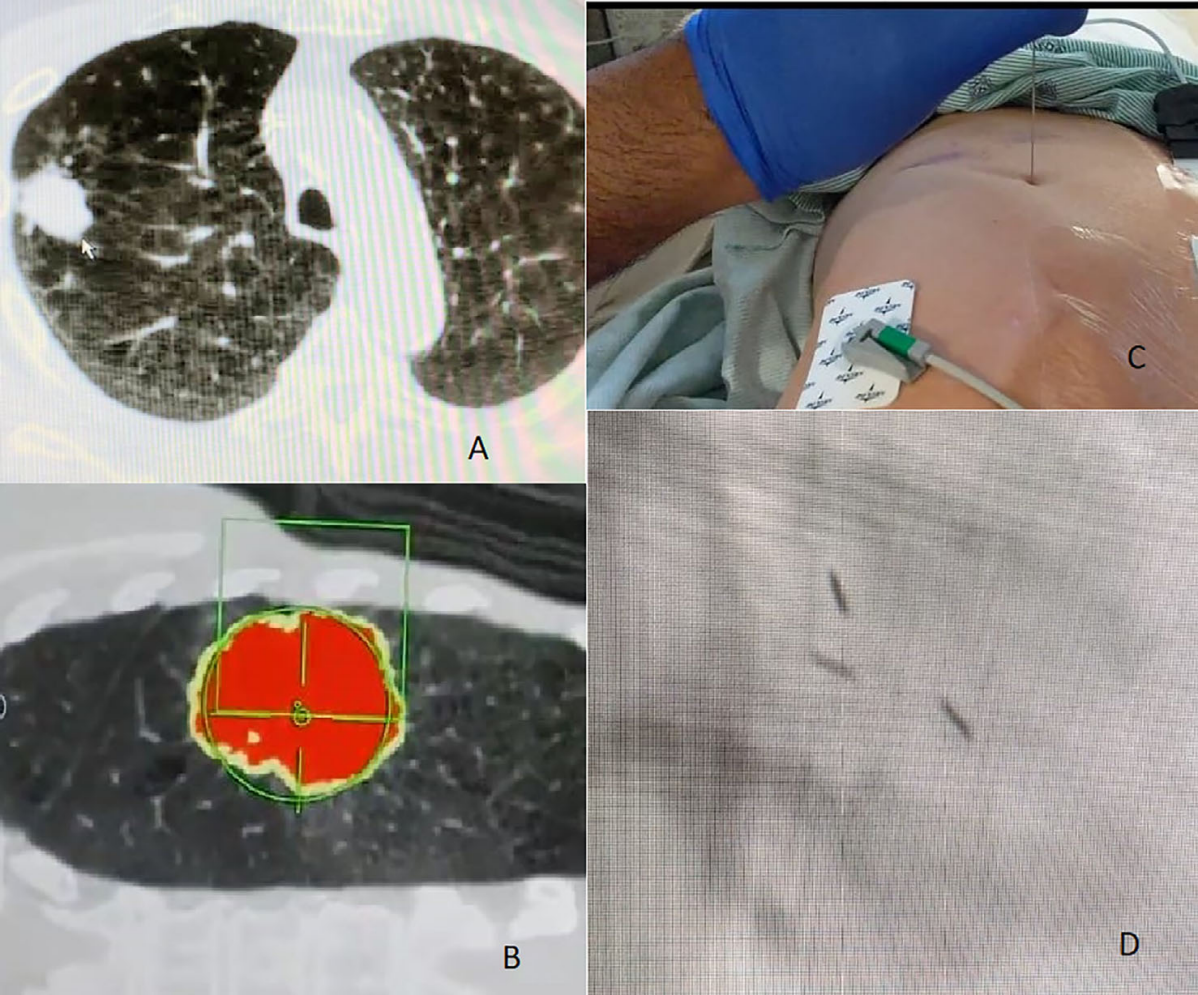
Example of an electronic navigational bronchoscopy (ENB) needle‐guided fiducial placement. A: Tumour seen on computed tomography. B: Biopsy planning with ENB needle. C: Fiducial placement through ENB needle trocar. D: Fiducials seen in the tumour on fluoroscopy

Figure 1 shows an example of an ENB needle‐guided fiducial placement.

### Results

Six patients were identified in total. Three patients had fiducials placed with US guidance and three with ENB‐needle.

**FIGURE 2:**
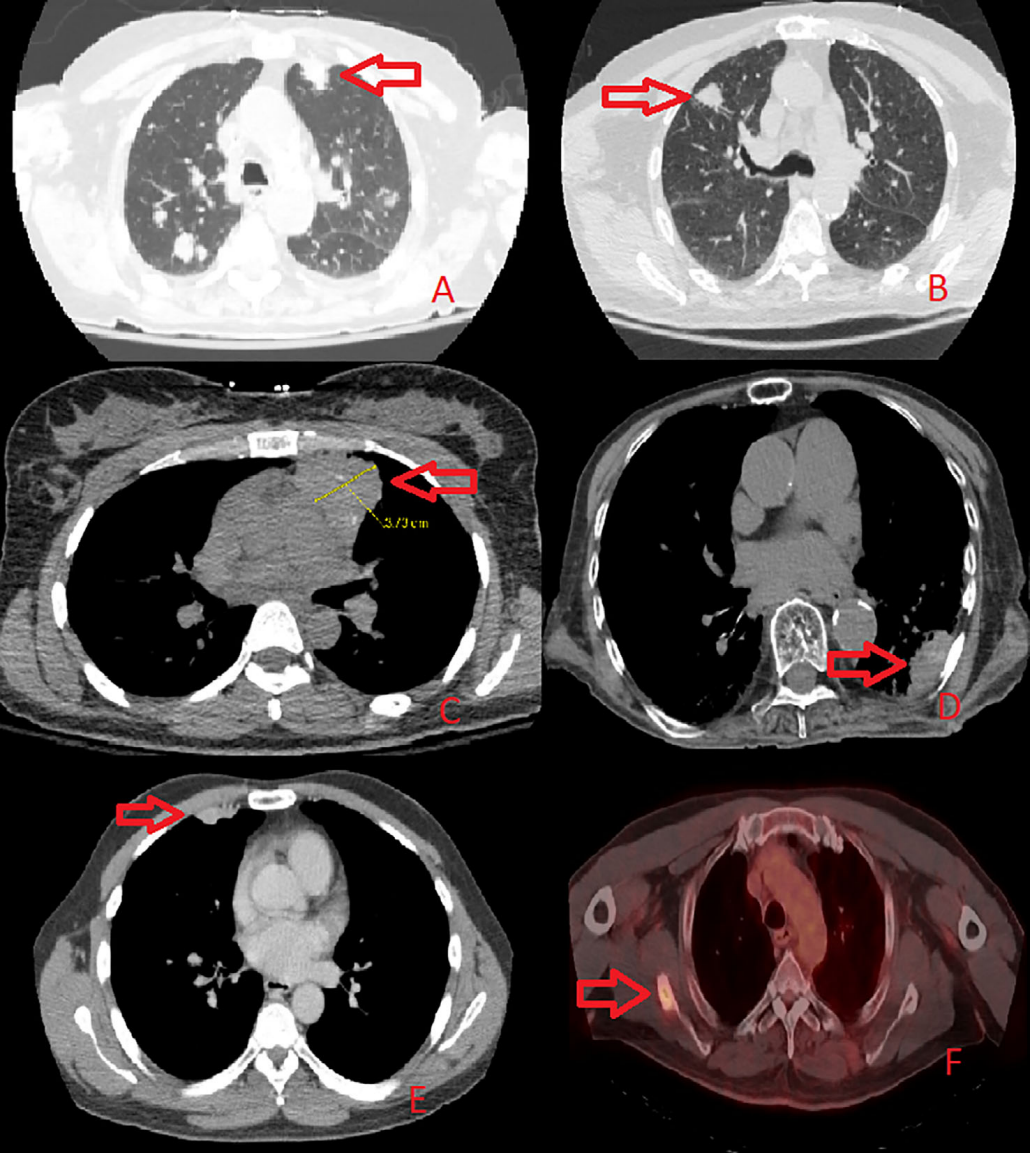
Sample computed tomography images of the six masses where fiducials were implanted (A: Patient 1; B: Patient 2; C: Patient 3; D: Patient 4; E: Patient 5; F: Patient 6)

Figure 2 shows sample images of the CT scans for the six patients.

Table 1 shows the characteristics of the patients, procedures performed and outcomes.

**TABLE 1:** Demographics, procedures performed, characteristics of masses and outcomes at radiosurgery planning

Age	Sex	Underlying malignancy	Smoking history	Location of mass	Distance between mass and pleura (cm)	Number and method of fiducial placement	Fiducials retained at radiosurgery planning	Distance between fiducials and tumour (cm)	Other interventions during the same procedure
44	F	Thymoma	Non‐smoker	Left upper lobe	0	2 (ENB needle)	2	0	Navigational bronchoscopy + convex EBUS
84	M	Squamous cell lung cancer	45 pack‐years	Right upper lobe	1.3	2 (ENB needle)	2	1.1	Navigational bronchoscopy
67	F	Breast cancer	Non‐smoker	Left upper lobe	0	2 (ENB needle)	2	0	Navigational bronchoscopy
77	F	Choriocarcinoma	5 pack‐years	Left upper lobe	0	2 (US)	1	0	
54	F	Synovial sarcoma	10 pack‐years	Right lower lobe	0	4 (US)	3	0	Bronchoscopy with radial EBUS
58	M	Lung cancer	40 pack‐years	Right side of scapula	N/A	3 (US)	2	0.1	

Abbreviations: EBUS, endobronchial ultrasound; ENB, electronic navigational bronchoscopy; F, female; M, male; NSCLC, Squamous cell lung cancer; US, ultrasound.

In total, 12 of 15 fiducials were retained and three migrated (80% retention rate). No complications were noted during the procedure or in relation to the migrated fiducials. All patients successfully proceeded to radiation treatment and no additional procedures were required.

## DISCUSSION

SBRT has become one of the mainstays of treatment for thoracic malignancies. While surgical resection of localized tumours is still considered the standard of care, many patients are not surgical candidates, and SBRT may be the only possible treatment modality in such cases. There are no randomized controlled trials comparing SBRT to surgical resection in operable patients. However, pooled analysis of incomplete randomized trials (closed for poor accrual) suggests a favourable outcome with SBRT,^1^ hence the importance of fiducial marker placement to facilitate this technique.

Our case series shows that fiducial placement using US or ENB needle guidance by the interventional pulmonologist, when feasible, is safe and effective, and leads to successful stereotactic treatment.

The option of percutaneous fiducial placement using US guidance has been used for several years, mainly described for abdominal malignancies,^6^ but also in thoracic locations. This modality allows direct real‐time visualization of lung masses adjacent to the pleura and placement of fiducials directly into the tumour. It can be done with no prior preparation and using only topical anaesthesia. Its main limitation is that any normal lung parenchyma between the chest wall and the mass is radio‐opaque and can impair visualization of the mass, hence limiting this technique to chest wall tumours and lung tumours in direct contact with the pleura.

In the case of ENB needle guidance, deeper masses may be targeted. The software allows the physician to see where the tip of the needle is and how close it is to the mass. Despite the fact that it relies on ‘virtual’ data and quality of synchronization with a CT scan (making it less accurate), this matters less in fiducial placement as they only need to be placed in proximity to the mass (fiducials placed 1 cm away from the mass may be used effectively for radiation therapy). As was mentioned in the Results section, using these two methods allowed placement of fiducials inside the mass in four cases, 0.1 cm from the mass in one case and 1.1 cm from the mass in one case. All our patients proceeded to radiation and none required additional procedures prior to therapy.

We also note, for the ENB needle method, the need to perform a special scan on the day of the procedure, as well as a bronchoscopy to calibrate and synchronize the data to the respiratory phase of the patient in real time. However, it is important to consider that a bronchoscopic procedure is often needed for these patients (to obtain diagnostic material, sample lymph nodes in the mediastinum and hila for staging purposes, etc.). Transthoracic placement of fiducials using the ENB needle system can be done during the same procedure (especially if suspicion of malignancy is high and if the plan is to proceed with radiosurgery even if biopsies are non‐diagnostic), thereby saving time and effort for the patient and reducing overall costs.

Fiducial placement techniques are normally considered successful when the patient proceeds to SBRT without additional procedures. In that sense, our success rate was 100%. In addition, the overall rate of retention of fiducial markers in these patients was excellent, and no complications were noted. This profile compares favourably to that of CT‐guided fiducial placement, where rate of retention is around 90% but with a high rate of complication (33% on average, up to 67% in some reports).^7^, ^8^, ^9^

Larger studies are needed to confirm these findings, but our data with regard to these interventions are very promising.

In conclusion, placement of fiducials transthoracically by the interventional pulmonologist using US or ENB needle guidance is a safe and effective technique, and can be combined with other diagnostic or therapeutic interventions in a single procedure.

## CONFLICT OF INTEREST

None declared.

## ETHICS STATEMENT

Appropriate written informed consent was obtained for publication of this manuscript and accompanying images.

## AUTHOR CONTRIBUTIONS

Drs Wissam Abouzgheib, Ziad Boujaoude and Dany Gaspard conceived the idea and performed all procedures. Dr Gregory Kubicek performed the radiation oncology evaluation and measured the distance between the fiducials and tumours. All authors contributed to the writing of the manuscript.
